# Probiotics: How Effective Are They in the Fight against Obesity?

**DOI:** 10.3390/nu11020258

**Published:** 2019-01-24

**Authors:** Kiran Mazloom, Imran Siddiqi, Mihai Covasa

**Affiliations:** 1 Department of Basic Medical Sciences, College of Osteopathic Medicine, Western University of Health Sciences, Pomona, CA 91766, USA; kiran.mazloom@westernu.edu (K.M.); imran.siddiqi@westernu.edu (I.S.); 2 Department of Health and Human Development, University of Suceava, Suceava 720229, Romania

**Keywords:** microbiota, inflammation, adiposity, lactobacillus, bifidobacterium

## Abstract

Obesity has been associated with structural and functional changes in the gut microbiota. The abundance in, and diversity of, certain bacteria may favor energy harvest and metabolic pathways leading to obesity. Therefore, gut microbiota has become a potential target that can be manipulated to obtain optimal health. Probiotics have been shown to influence the composition of the gut microbiota, improve gut integrity, and restore the microbial shifts characteristic of obesity. Based on physical and biochemical parameters, metabolic and inflammatory markers, and alterations in gut microbe diversity, animal studies revealed beneficial results in obese models whereas the results in humans are sparse and inconsistent. Thus, the purpose of this review is to present evidence from animal studies and human clinical trials demonstrating the effects of various probiotic strains and their potential efficacy in improving obesity and associated metabolic dysfunctions. Furthermore, the review discusses current gaps in our understanding of how probiotics modulate gut microflora to protect against obesity. Finally, we propose future studies and methodological approaches that may shed light on the challenges facing the scientific community in deciphering the host–bacteria interaction in obesity.

## 1. Introduction

Obesity is now considered a worldwide pandemic affecting approximately 1 in 3 individuals [1]. Despite significant efforts in the past decade to control the incidence of obesity, progress has been slow in understanding the etiology and the various mechanisms mediating its development that may lead to the identification of viable therapeutic approaches for treatment. Among the array of factors and their complex interactions that play a role in obesity, new, accumulating evidence show that the gut microbiota is an important contributor. The gut microbiota represents the sum of all bacteria that are present in the gastrointestinal (GI) tract starting from the oral cavity and increasing in its density along the small and large intestine [2]. Thus, for the purpose of this review, we will refer to the “microbiota” as the physical bacteria present in the GI tract and “microbiome” as the set of genes that form the bacterial strains.

A large body of evidence has described several possible mechanisms by which the gut microbiota can contribute to, and/or influence, obesity. Although so much is still unknown and debatable, thus far there is a general consensus that the gut microbiota is implicated in obesity through dietary carbohydrate fermentation, lipogenesis, excess energy storage, and several other pathways including a vast array of metabolites, hormones and neurotransmitters, some of which are known to control food intake and the regulation of energy balance [3,4,5]. Furthermore, there is convincing literature demonstrating that the composition and diversity of the gut microbiota is altered in obese rodents and humans when compared to lean counterparts. For example, gut microbiota composition is modified in obesity resulting in an enrichment or reduction in the proportions of specific bacterial groups. Similarly, gut microbiota gene richness is also affected in obesity with studies showing a 20–40% decrease in the diversity of bacteria [6,7]. These findings suggest that restoration of the compositional profile and richness of the gut microbiota may result in rescuing the obese phenotype and associated metabolic defects. One way of accomplishing this is through the use of prebiotics, probiotics, and synbiotics. Therefore, in this review, we will discuss the role of probiotics, their effects and mechanisms implicated in the modulation of gut microbiota and its subsequent impact on obesity development using comparative evidence from both preclinical and human clinical studies. Furthermore, we will present and discuss current gaps in our understanding of how probiotics-induced changes in gut microbiota profile may mitigate host metabolic dysregulations accompanying obesity. Finally, we will propose future studies and methodological approaches that may shed light on the challenges facing the scientific community in deciphering the host–bacteria interaction in obesity.

## 2. Gut Microbiota Composition and Function

The intestinal gut microbiota is a complex organ system that is critical for the health of the host. In recent years, the gut microbiome, that encompasses the genes of microbial cells, has been intensely scrutinized through genetic and molecular techniques of identification, including 16S ribosomal RNA gene sequencing, to determine which microorganisms reside in the gut and how they function [8]. There are approximately 10^1^ to 10^3^ cfu/mL of bacteria in the proximal small intestine, 10^4^ to 10^7^ cfu/mL of bacteria in the distal small intestine, and 10^4^ to 10^11^ cfu/mL of bacteria in the colon [2]. The gut microbiome consists of three main phyla: Bacteroidetes (*Porphyromonas, Prevotella, Bacteroides*), Firmicutes (*Ruminococcus, Clostridium, Lactobacillus* and *Eubacteria*), and Actinobacteria (*Bifidobacteria*) [9] with the majority of the intestinal flora being represented by *Bifidobacterium* and *Bacteroides* [10]. These microorganisms have important protective, structural, and metabolic functions. For example, the commensal bacteria in the gut microbiome protect the host by displacing harmful bacteria, competing with pathogens for nutrients, and producing anti-microbial factors. In addition, these bacteria provide the host with structural functions, such as developing the immune system, inducing immunoglobulin A (IgA), and reinforcing the mucosal barrier. Furthermore, the commensal bacteria provide metabolic functions to benefit the host by synthesizing vitamin K, folate, and biotin, among other as well as participating in the absorption of magnesium, calcium, and iron ions. These bacteria also metabolize dietary compounds and ferment non-digestible dietary foods resulting in the formation of short-chain fatty acids (SCFAs) [2].

## 3. Gut Microbiota and Obesity

The link between gut microbiota and obesity has been suggested by the early pioneering studies showing that adult mice devoid of gut microbiota (i.e., germ free) acquired a 60% increase in body fat content after they were recolonized with a healthy cecal microbiota [11,12]. The initial mechanism thought to be responsible for such an increase in body fat was attributed to the ability of microbiota to extract energy from food constituents and regulate the energy balance of the host. Degradation of dietary polysaccharides and fiber by *Bacteroides* and *Firmicutes* in the gut results in the production of SCFAs, such as propionate, acetate, and butyrate. Propionate is an important energy source for the host via de novo synthesis of lipids and glucose in the liver [3,4,5,13]. Acetate is used in peripheral tissues as a substrate for cholesterol synthesis [4] while butyrate represents a rich energy source for the epithelial cells that line the colon [14]. Furthermore, microbiota is involved in the control of energy balance, food intake, and satiety via gut peptide signaling, through hormonal effects in the blood or by modulating the nervous system directly. The appropriate balance of these regulatory peptides may be disrupted if the microbiota composition is altered, as evidenced by germ-free mice having increased levels of pro-obesity peptides like neuropeptide-Y and reduced levels of anti-obesity peptides [15]. The gut is also involved in nutrient sensing, with metabolic products from bacteria activating enteroendocrine cells (EEC) through paracrine signaling from enterocytes [16]. In vitro and in vivo studies have demonstrated that SCFAs may be used as main energy source, but they also serve as signaling molecules that can activate G-protein coupled receptors (GPRs), including GPR43 (also known as free fatty acid receptor 2) in adipose and intestinal tissues [17]. In adipose tissue, SCFAs bind to GPR43, thus promoting adipogenesis and increasing energy expenditure [18]. In intestinal tissue, SCFAs bind to GPR43 leading to secretion of anorexigenic peptides, including glucagon-like peptide-1 (GLP-1) and peptide YY (PYY), resulting in improved glucose tolerance and increased energy utilization. Additionally, increased production of selected SCFAs is associated with high levels of ghrelin and insulin [16]. In particular, butyrate is involved in energy regulation by stimulating L cells, a subpopulation of EEC, to secrete GLP-1. GLP-1, a peptide involved in satiety and insulin secretion, has been found in lower quantities in obese compared to lean individuals [19]. Similarly, PYY, also produced by the intestinal L cells, is important for satiety, increasing in concentration during the postprandial period [20]. As such, administration of PYY-3-36 in obese individuals results in a significant reduction of food intake [21]. Thus, GLP-1 and PYY act as appetite suppressants and are potent mediators of the gut–brain axis, which facilitate important cross-talk regarding energy homeostasis, digestion, and appetite [22]. They act to decrease intestinal motility, gastric emptying and regulate glucose homeostasis and energy utilization [23].

The orexigenic gastric peptide hormone, ghrelin, is negatively correlated with *Bifidobacterium, Lactobacillus*, and *Blautia coccoides/Eubacterium rectale*, and is positively correlated with *Bacteroides and Prevotella*. Ghrelin has several functions including stimulation of gastric emptying, appetite stimulation, glucagon secretion, and inhibition of insulin secretion and thermogenesis [16]. It has been shown that cells which produce ghrelin have GPR43 receptors, but it is not clear yet if gut metabolites directly stimulate these receptors, resulting in ghrelin secretion. By contrast, the anorexigenic hormone leptin is positively correlated with *Bifidobacterium* and *Lactobacillus.* Although it is not clear whether these are causal relationships, it is thought that leptin can modulate gut microbiota by stimulating mucin production, which may favor differential bacterial growth [24]. Lastly, it should be noted that microbiota composition can affect EEC counts and their respective receptor expressions, as the *Firmicutes* to *Bacteroidetes* ratio was positively correlated with GPR43 expression in obese mice [25]. In summary, SCFAs binding to GPR43 in both adipose and intestinal tissues regulate obesity and energy accumulation. This mechanism helps maintain energy homeostasis and may be used as a tool in the treatment of metabolic diseases [17].

The gut microbiota not only enhances lipogenesis, but also reduces levels of fast-induced adipose factor (FIAF), also called Angiopoietin-like 4 protein (ANGPTL4) a lipoprotein lipase (LPL) inhibitor produced by the liver, intestine, and adipose tissue and a main regulator of metabolism and adiposity (see [26,27]). Increased intake of a high carbohydrate and fat diet can lead to dysbiosis and increased triglyceride deposition in adipose tissue which is associated with decreased FIAF expression [12]. This, in turn, leads to enhanced adipocyte LPL activity resulting in increased uptake of fatty acids, increased fat storage and ultimately obesity [11]. As a result, FIAF serves as a protective mechanism against diet-induced obesity. However, whether gut microbiota influences FIAF levels in obesity is still unclear since high fat diet-induced obesity in germ free mice only increases mRNA expression of FIAF in the intestine but not in the circulation [28]. In addition, gut microbiota inhibit the activity of AMP-activated protein kinase (AMPK), an important liver and skeletal muscle enzyme with a role in cellular energy homeostasis and obesity. When energy expenditure is low, AMPK is decreased resulting in less activation of enzymes involved in beta oxidation, including acetyl CoA carboxylase and carnitine palmitoyltransferase I, thus leading to obesity [29]. This explains why germ-free mice fed a Western-type diet have increased levels of phosphorylated AMPK promoting fatty acid oxidation [29]. Therefore, gut microbiota suppresses AMPK activity leading to heightened cholesterol and triglycerides synthesis, lipogenesis, excess fat accumulation and obesity [30].

Obesity has also been linked to low-grade inflammation due to the failure of intestinal epithelial membrane receptor proteins that play a sensory role in the gut [31,32,33]. This causes increased gut permeability, reduced expression of tight junction proteins leading to bacterial fragments, such as lipopolysaccharides (LPS) to diffuse through the gut and into the bloodstream, resulting in metabolic endotoxemia. LPS then combines with pattern recognition receptor CD14, which, together, are recognized by toll-like receptor-4 (TLR4), a major component of the innate immune system that maintains intestinal homeostasis. Individuals who consume a high-fat diet have increased plasma LPS levels [34,35] that stimulate cells through TLR4 leading to the development of low-grade inflammation observed in obesity [36,37]. Increased LPS plasma concentration either via high fat feeding or experimentally induced (i.e., through infusion) results in metabolic changes and systemic inflammation [38]. These changes were associated with a significant reduction in the population of *Lactobacillus spp., Bifidobacterium spp., and Bacteroides–Prevotella spp* in the gut. On the other hand, administration of *Bifidobacteria* to rodents with thermal injury improved gut integrity and metabolic endotoxemia [39]. 

A further link between microbiota and obesity lies in the ability of *Firmicutes* and *Actinobacteria* to produce conjugated linoleic acid (CLA). Altered production of this fatty acid is concerning in the context of obesity because CLA has been shown to have several anti-obesity effects, including increased energy metabolism, energy expenditure, and lipolysis as well as decreased adipogenesis and lipogenesis [40]. Additionally, studies have demonstrated that CLA decreases de novo lipid synthesis and induces adipocyte apoptosis [41]. Apoptosis of adipose tissue is associated with the induction of TNF-alpha and uncoupling protein-2. As the name implies, uncoupling protein-2 “uncouples” electron transfer across the inner mitochondrial membrane, which results in the thermal dissipation of energy as opposed to conversion to the energy storage molecule adenosine triphosphate (ATP) [42]. It is thought that CLA mediates some of its effects by displacing arachidonic acid from the phospholipids contained in cell membranes, thereby decreasing synthesis of eicosanoids like prostaglandins and leukotrienes, well known players in inflammation. CLA has signaling functions as well, including activation of transcription factors and peroxisome proliferator-activated receptors (PPARs), which have downstream effects on lipid metabolism and immune function [43]. Lastly, when administered to mice, CLA enhanced sympathetic nervous system activity which led to increased energy metabolism and reduced adipose tissue [44].

## 4. Gut Microbiota Strains and Obesity 

The ratio of the two prevalent bacterial phyla in the gut microbiota, Firmicutes and Bacteroidetes that have been shown to produce SCFAs from non-digested dietary compounds has been proposed as a marker for obesity. For example, obese individuals tend to have higher proportion of Firmicutes and decreased proportion of Bacteroidetes [45]. Even though Bacteroidetes do possess the genes to produce enzymes involved in lipid and carbohydrate metabolism, Firmicutes possess significantly more resulting in increased fermented end products, including SCFAs [46,47]. Indeed, several experiments have confirmed an increase in the ratio of Firmicutes to Bacteroidetes, also known as the F/B ratio, in obese individuals [48,49,50,51]. In particular, one study has concluded that the prevalence of *Bacteroides fragilis*, a commensal Gram-negative bacteria, is implicated in obesity [52]. However, the importance of the F/B ratio remains controversial as other studies have shown no correlation between the F/B ratio and obesity and that no significant differences between the two phyla are present in obesity [49,53,54]. Similarly, another recent study showed that *Bacteroides vulgatus*, one of the most abundant bacteria in the human gut and a pathobiont was strongly associated with inflammation, insulin resistance, and altered metabolism. In addition, a reduction in several bacteria from the phylum Firmicutes, such as *Blautia, Faecalibacterium*, and others in the Clostridiales order, correlated with increased trunk-fat [55]. Furthermore, several studies focused on the relationship between non-bacterial, methanogenic archaea and obesity. In particular, a reduction in *Methanobrevibacter smithii* has been implicated in obesity [56]. *M. smithii* encourages fermentation and metabolism by using hydrogen, an end product of fermentation. A reduction in *M. smithii* may lead to decreased metabolism and an increased risk of obesity [45]. Other bacteria such as *Lactobacillus* are also present in high quantities in obese and overweight children, whereas high levels of *Bifidobacterium* were found in lean children [52]. Lastly, a high prevalence of *Faecalibacterium prausnitzii*, the most abundant Gram-positive commensal bacteria present in the gut, has been linked with obesity [57]. Therefore, current evidence shows microbiota compositional differences in obese compared to healthy, non-obese organisms.

The abundance or richness of bacterial genes has also been associated with obesity. For example, low gene richness or counts (LGC) correlated with increased trunk-fat and obesity. In a large study involving 61 severely obese women, it was found that 75% of the subjects had low gene counts [55] compared to only 23–40% when subjects were overweight or moderately obese [6,7]. In addition, certain metabolites and the proteins involved in their metabolism were associated with low microbial gene richness (MGR). For example, as trunk fat mass increases, MGR decreases along with the metabolite, 3-methoxyphenylacetic acid. This acid is a product of polyphenol and flavonoid fermentation and may have beneficial effects on the gut microbiota. As such, a reduction in histidine and enzymes involved in histidine production and degradation has also been implicated in obesity. Gamma-aminobutyric acid (GABA), a precursor to histidine production that is linked to downregulation of pro-inflammatory cytokines, is one such pathway that is negatively affected in obesity leading to further inflammation [55].

## 5. Modulation of Gut Microbiota by Probiotics 

Numerous studies have shown that the gut microbiota not only has a key role in the physiology of the host but also plays a modulatory role in obesity. This suggests that manipulation of gut microbiota through dietary or other means may confer beneficial effects by restoring gut functional integrity and reverse dysbiosis that is characteristic of obesity. Such an approach is highly desirable, as it would decrease treatment costs and significantly diminish the risk of harm to the patient compared to more drastic and invasive interventions currently used to treat obesity such as bariatric surgery. In this regard, probiotics have been extensively studied and widely thought to be the intervention of choice in manipulating gut microbiota composition. 

Defined by the World Health Organization (WHO) and by the Food and Agricultural Organization, as non-pathologic living microorganisms, probiotics have been shown to confer health benefits to the host when administered in adequate amounts. The term “probiotics” comes from the Greek word meaning “for life” [58]. Elie Metchnikoff was the first to observe the beneficial effects of fermented milk containing lactic acid bacteria on the longevity of Bulgarian populations in the early 20th century. Further building off Metchnikoff’s research on the beneficial effects of bacteria, Henri Tissier at the Pasteur Institute in France, administered *Bifidobacteria* to infants suffering from diarrhea after discovering *Bifidobacteria* in the gut microbiota of human milk-fed babies [59]. Among the most well-studied probiotics are lactic acid bacterial strains belonging to *Bifidobacteria, Lactobacilli*, which have an established safety record and have been given GRAS (generally recognized as safe) status by the United States Food and Drug Administration. Other bacterial probiotics that are still being explored include the genera *Bacillus, Escherichia*, and *Proprionibacterium* [60]. Some general characteristics that serve in the identification of probiotic candidates includes features that would facilitate colonization such as: tolerance to low pH, resistance to bile salts, and adhesion to host gut epithelium [61]. Probiotics interact with the host either in transit or by colonization with several downstream mechanisms underlying their health-promoting effects. Some of the mechanisms currently under study include modification of the gut microbiota composition, strengthening of the gut epithelial barrier, competitive adherence to the gut mucosa, production of health promoting and antimicrobial substances, and modulation of the immune system to confer advantages to the host.

### 5.1. Enhancement of Epithelial Barrier Integrity 

The intestinal epithelial barrier serves as a vital defense mechanism for the host. This barrier consists of a mucous layer, antimicrobial peptides, secretory IgA, and epithelial junction adhesion complex [62]. If the integrity of the gut epithelial barrier is compromised, various antigens may access the submucosa, triggering an inflammatory response, which is seen in a range of pathologies from inflammatory bowel disease to obesity [63]. Although not completely understood, the administration of probiotics has been shown to aid in the functionality of the intestinal barrier [64]. *Lactobacilli* modulate expression of numerous genes encoding adherence junction proteins like E-cadherin, B-catenin in a T84 cell barrier model [65]. Additionally, *Escherichia coli* Nissle 1917 (EcN1917) not only prevented derangement of the mucosal barrier by a pathogenic *E. coli*, but also restored integrity in T84 and Caco-2 cells. This effect was mediated by increased expression and repositioning of tight junction proteins of the zonula occludens (ZO-2) and protein kinase C (PKC), which led to the reconstruction of tight junction complex [66]. Some of the major macromolecular components of the epithelial mucus include mucin glycoproteins, and may have implications in the development of metabolic syndrome. Probiotics that can promote mucous secretion may improve gut barrier function and exclude pathogens. One example is the probiotic VSL3 administered to rats for 7 days, which had a 60-fold increase in *MUC 2* expression as well as an increased secretion of mucin [67]. Also, VSL3 (a mixture of probiotics and prebiotics) co-defends the epithelial barrier and increases tight junction protein expression through activation of p38 and extracellular kinase pathways [68].

### 5.2. Enhanced Adhesion to Intestinal Mucosa 

Adequate adhesion to the intestinal mucosa underpins colonization of the host gut and may be essential in interactions between probiotics and the host [69]. Several *Lactobacillus* proteins have been demonstrated to promote mucous adhesion. Additionally probiotics in this genera display surface adhesins that facilitate attachment to the mucous layer in the host gut. One such protein is MUB (mucus-binding protein), produced by *Lactobacillus reuteri* [70]. *Bifidobacterium animalis* subp. *lactis* also has surface proteins that interact with human enterocytes, and have an array of functional implications, including facilitating colonization through the degradation of extracellular matrix of cells or by enabling close contact with the epithelial surface [71]. 

### 5.3. Production of Health-Promoting and Antimicrobial Substances 

Certain *Bifidobacteria* and *Lactobacilli* have been shown to produce health-promoting conjugated linoleic acid (CLA), which is a known anti-carcinogenic agent. In diet-induced obese mice, CLA-producing *L. plantarum* had significant anti-obesity effects [72]. Supporting the importance of CLA production, another study used a murine model to demonstrate that oral administration of CLA-producing *Bifidobacteria* and *Lactobacilli* positively modulated fatty acid composition of liver and adipose tissue of the host [73]. In addition to producing molecules that promote beneficial functions in the host, many lactic acid bacteria (LAB) produce small antimicrobial peptides (AMPs), including bacteriocins that may ward off pathogenic bacteria. Bacteriocins vary among different species but the central mechanisms entail the destruction of target cells by pore formation and inhibition of cell wall synthesis [74].

### 5.4. Exclusion of Pathogenic Microbes

Another pathway by which probiotics exert their beneficial effects is the competitive exclusion of pathogenic microbes in the gut. Although there are several ways that bacteria may confer these effects, some important mechanisms include the creation of a hostile microenvironment, eliminating bacterial receptor sites, producing antimicrobial substances, and depleting available nutrients required for pathogen survival [75]. *Lactobacilli* and *Bifidobacteria* have been shown to inhibit a variety of pathogens, including *E. coli, Salmonella, Helicobacter pylori, Listeria monocytogenes* and *Rotavirus* [76]. One way that probiotics exhibit these effects is through steric hindrance at enterocyte pathogen receptors, limiting the attachment of pathogenic bacteria [77]. Lastly, some probiotics can modify their local environment by producing antimicrobial substances like lactic acid and acetic acid, creating a deleterious microenvironment for pathogens [78].

### 5.5. Modulation of Host Immune System

One of the major mechanisms of probiotic action is through the regulation of host immune response and cytokine profile [79,80]. An important part of the interplay between probiotics and the host immune system is through microbe-associated molecular patterns, such as cell wall and cytoplasmic membrane-anchored molecules like polysaccharides, peptidoglycans, lipoproteins, lipoteichoic acids, which are recognized by pattern recognition receptors expressed in epithelial, and immune cells of the host (ex. TLRs). These microbe-associated molecular patterns vary considerably in their chemical structure among probiotic strains even within the same genera, and may in part explain the strain specificity of probiotics. One such example is that *Lactobacilli* species differ widely in their ability to trigger TLR2 signaling [81]. 

A number of animal studies and human clinical trials examined the efficacy of probiotics as a useful treatment for obesity through the modulation of the gut microbiome, and their outcomes are reviewed in the following sections.

## 6. Probiotics in Animal Studies 

### Lactobacillus

Over the past several years, promising preclinical studies investigating the effects of probiotic supplementation on the development of obesity have emerged. A majority of the studies reviewed here focused on intervention with *Lactobacillus* spp. and have demonstrated considerable anti-obesity effects and the potential for probiotic based therapies in the treatment and prevention of obesity [82]. One of the early studies conducted over an eight-week period showed that *Lactobacillus rhamnosus* PL60 reduced body weight without reducing energy intake, and significantly reduced white adipose tissue. Signals of apoptosis and uncoupling protein-2 (UCP-2) mRNA levels were increased in adipose tissue, while fatty acid synthase and serum leptin were simultaneously reduced [83]. The authors attributed these effects to the production of t10, c12-conjugated linoleic acid by the probiotic [83]. In a follow-up study, t10, c12-CLA-producing *L. plantarum* PL62 reduced epididymal, inguinal, mesenteric, and perineal white adipose tissue mass, while also significantly lowering body weight and blood glucose levels in diet-induced obese mice [72]. In order to investigate the insulin-sensitizing mechanisms associated with *Lactobacillus rhamnosus* GG (LGG), mice fed a high-fat diet (HFD) were given oral LGG for 13 weeks, which resulted in attenuated weight gain, and improved insulin sensitivity. Concurrently, LGG increased the expression of fatty acid oxidative genes in the liver while it decreased gluconeogenic genes. This resulted in reductions in lipid accumulation by stimulating adiponectin secretion and downstream activation of AMPK, an enzyme involved in controlling the energy status of cells [84]. *L. rhamnosus* NCDC 17 improved oral glucose tolerance test, biochemical parameters and oxidative stress in diabetic rats [85]. *L. rhamnosus* alone or in combination with any of the herbal preparations (Aloe vera/Gymnema sylvestre powders, 1% *w/w*) seems to show anti-obesity and anti-inflammatory properties in mice fed the high-fat diet [86]. Thus, modulation of gut microbiota by probiotic strains containing *Lactobacillus rhamnosus* can have beneficial effects on body weight, glucose and fat metabolism, insulin sensitivity and chronic systemic inflammation.

Several studies have tested the effects of the gram positive *L. plantarum*, a widespread member of the genus *Lactobacillus* and with the largest genome from the lactic acid bacteria group, in obesity. For example, Park et al. demonstrated that *L. plantarum* Q180 administered to mice on HFD reduced body weight gain, and concurrently decreased triglycerides, serum leptin, aspartate aminotransferase (AST), and epididymal fat weight [87]. Similarly, a 4-week study with HFD rats supplemented with a high protein whey beverage containing kimchi-derived *Lactobacillus plantarum* DK211 prevented body weight gain, and body fat accumulation. The treatment group also saw a decrease in organ weight, total cholesterol, LDL cholesterol, triglycerides, blood glucose, and serum insulin, leptin, as well as ghrelin compared to the HFD group [88]. Similarly, *L. plantarum* LG42 administered to male C57BL/6J mice on a HFD for 12 weeks had significant anti-obesity effects such as lowered body weight, with a significant reduction in epididymal and back fat; decrease in hepatic triglyceride, serum insulin, and leptin levels; increased mRNA expression of PPARα and CPT-I; decreased levels of acetyl-CoA carboxylase, SREBP-1, and LXRα compared to control; reduced expression of PPARγ and its downstream genes [89]. Furthermore, when tested for its immuno-modulatory role, *L. plantarum* TN8 showed protective effects on lipid, hepatic, and renal profiles in obese rats [90]. This *Lactobacillus* strain not only improved body weight, but also had other beneficial anti-obesity effects by increasing IL-10 secretion while decreasing other pro-inflammatory cytokine production [90]. Along the same lines, administration of another *L. plantarum* strain, Ln4, a lactic acid bacteria isolated from fermented foods, reduced weight gain and epididymal fat mass and lowered plasma triglycerides in mice fed a high-fat diet [91]. Protein levels of adipokines such as C-reactive protein (CRP), insulin-like growth factor binding proteins-3 (IGFBP-3), and monocyte chemoattractant protein-1 (MCP-1) were also decreased in white adipose tissue of mice treated with Ln4. In addition, this probiotic strain induced changes in expression of several hepatic genes involved in regulation of glucose and lipid metabolism including increased IRS2, Akt2, AMPK, LPL, and reduced CD36, resulting in reduced insulin resistance, improved glucose tolerance and insulin response. Together, these results point to Ln4 as a potential therapeutic probiotic agent for the treatment of metabolic disorders. Finally, *L. plantarum* strain No. 14 (LP14) given to HFD female mice reduced body weight gain, mean adipocyte size, white adipose tissue weight, total cholesterol, and leptin after 11 weeks of administration. However, in a separate experiment, LP14 had no influence on serum triacylglycerol accumulation following olive oil administration in Triton WR1339-treated mice, which suggest that dietary fat absorption is unaffected by LP14 and its beneficial effects are achieved through an alternate mechanism [92]. However, Lactobacillus plantarum (LP625) alone and in combination with herbs (Aloe vera and Gymnema sylvestre) decreased body and epididymal fat weight and reduced expression of pro-inflammatory cytokines.in mice fed the high-fat diet [93]. Thus, based on the studies presented, *L. plantarum* has proved, at least in the animal studies, an effective probiotic with anti-inflammatory and anti-obesity effects. 

Several other *Lactobacillus* strains such as *Lactobacillus gasseri* and *paracasei* have been studied for their anti-obesity and anti-inflammatory effects. For example, a 24-week long study found that administration of *Lactobacillus gasseri* SBT2055 to mice fed a 10% fat diet resulted in reduced expression of pro-inflammatory genes like CCL2 and CCR2 in adipose tissue, prevented body weight gain and fat accumulation, providing evidence that an improved inflammatory state could be mechanistically responsible for the aforementioned effects [94]. Similarly, rats fed skim milk with *L. gasseri* SBT2055 had a significant reduction in average adipocyte size, as well as decreased leptin and cholesterol [95]. Another *Lactobacillus gasseri* strain, BNR17 with promising anti-obesity effects reduced body weight, white adipose tissue weight, serum leptin, and insulin levels in mice fed a high carbohydrate diet when administered twice daily for 12 weeks [96]. Upregulation of fatty oxidation genes was also seen in addition to downregulation of genes involved in fatty acid synthesis, providing insights into the mechanistic basis for these positive results [97]. In an attempt to elucidate the role of the autonomic nervous system in the observed anti-obesity effects, *L. paracasei* ST11 (NCC2461) was administered intraduodenally. This led to an increase of sympathetic nerve activity in white and brown adipose tissue and, following intragastric injection, thermogenesis and lipolysis increased in brown and white adipose tissue, respectively. Overall, these changes indicate that the results of attenuated weight gain are, in part, due to excitation of the sympathetic nervous system, and a consequent lypolytic/thermogenic response [98]. In addition to examining the effects of single strains, several studies have used multiple, mixed bacterial strains. For example, one study assessed the effects of the prebiotic XOS, probiotic *L. paracasei* HII01, and synbiotic (1:1 combination of both) in HFD mice for 12 weeks. The results showed that the prebiotics, probiotics, and synbiotics, all significantly improved insulin sensitivity, and attenuated dyslipidemia [99]. Body weight and visceral fat, however, showed a significant reduction only in the pre-and synbiotic-treated groups. A significant decrease in LPS levels was seen equally in the treatment groups, indicating a reduction in metabolic endotoxemia. Pro-inflammatory cytokines, IL-6 and IL-1B, were also reduced. These benefits of *L. paracasei* HII01, XOS, and synbiotic combination could be due to the improvement of gut dysbiosis by lowering the amount of LPS-containing Enterobacteriaceae within the gut lumen, which mitigates the eventual LPS translocation into gut tissue and serum. Finally, combination of *L. rhamnosus* LGG with *L. sakei* NR28 for 3 weeks, produced a significant reduction in epididymal fat mass, as well as obesity-related biomarkers such as acetyl-CoA carboxylase, fatty acid synthase, stearoyl-CoA desaturase-1 in the liver of HFD mice [100]. Anti-hyperglycemic and hyperlipidemic effects of probiotics are strain-dependent as well as type of animal models [101]. Together, these preclinical studies show that treatment with selective probiotics or their combinations can result in amelioration of metabolic dysfunctions associated with obesity.

## 7. Changes in Microbiota Composition

The number and the ratio of microbial groups undergoing changes in the intestine following probiotic administration have been well documented. For example, there was a notable decrease in the ratio of Firmicutes to Bacteroidetes after *L. sakei* NR28 treatment in mice [100]. Similar alterations in the gut microbiota associated with reduced fat mass was found by Everard et al. who reported an increased proportion of Bacteroidetes, as well as decreased Firmicutes, Proteobacteria, and Tenericutes after obese/type 2 diabetic mice were treated with *Saccharomyces boulardii* [102]. Lastly, administration of *Lactobacillus salivarius* UCC118 Bac+ (bacteriocin-producing probiotic) to HFD mice over 20 weeks increased Bacteroidetes and Proteobacteria, and decreased Actinobacteria associated with suppressed body weight gain [103].

### 7.1. Bifidobacterium 

A 2012 study found that *Lactobacillus acidophilus* NCDC13 administration to diet-induced obese mice significantly increased *Bifidobacterium*, a genus that has been studied for its role in the amelioration of obesity, shifting the gut microbiota balance positively after 8 weeks, although its specific anti-obesity potential could not be established in mice fed a HFD [104]. Likewise, administration of *Bifidobacterium* probiotic strains increased fecal *Bifidobacterium pseudocatenulatum* SPM 1204, *B. longum* SPM 1205, and *B. longum* SPM 1207 in HFD rats [105]. This was associated with other anti-obesity effects such as reduced serum total cholesterol, HDL-C, LDL-C, triglycerides, glucose, leptin, AST/ALT, lipase, and reduction in harmful enzymatic activities done by β-glucosidase, β -glucuronidase, and tryptophanase [105]. When comparing the effects of B. L66-5 vs. L75-4 versus M13-4 vs. FS31-12 in HFD mice, it was shown that while all four strains reduced serum and liver triglycerides, only B. L66-5 reduced body weight [106]. In studies using another strain, *B. adolescentis* supplementation reduced body and fat weight of mice fed a high-fat diet [107]. *B. adolescentis* was further shown to reduce body weight gain, improve diet-induced nonalcoholic steatohepatitis, and decrease liver damage associated with the inhibition of lipid peroxidation, NFκB activation, and inflammation [108]. When comparing several *Bifidobacterium animalis subsp. lactis* I-2494 (BA), *L. paracasei* CNCM I-4270 (LC), and *L. rhamnosus* I-3690 (LR) Wang et al. showed that all individually attenuated weight gain and macrophage infiltration into epididymal adipose tissue, while also improving glucose-insulin homeostasis and hepatic steatosis of HFD mice after 12 weeks. Overall, all three strains shifted the HFD-disrupted gut back toward that of lean mice fed a chow diet, but found that while LC and LR increased cecal acetate, they did not affect lipopolysaccharide-binding protein in circulation. In contrast, BA did not increase acetate but decreased adipose and hepatic TNF-α expression, suggesting that *Lactobacillus* and *Bifidobacterium* attenuate obesity through a strain-specific, differential manner, and highlighted the importance of understanding specific mechanistic differences between probiotics [109].

### 7.2. Other Probiotic Strains

*Pediococcus pentosaceus* LP28 is a lactic acid, plant-derived bacteria, that was studied by Zhao and colleagues, who demonstrated that supplementation in HFD mice reduced body weight gain, liver lipids, and downregulated genes related to lipid metabolism (CD36, stearoyl-CoA desaturase 1 (SCD1), and PPARγ) [110]. Similarly, Cano et al. studied strains from the IATA-CSIC and Spanish Culture Collection (CECT) including *Bacteroides uniformis* CECT 7771 that was investigated for its utility in HFD-induced obesity. Supplementation resulted in decreased body weight gain, liver steatosis, triglycerides, and reduced dietary fat absorption. Additionally, this treatment ameliorated immune dysfunction seen in obese mice, which was evidenced by restored capacity of dendritic cells to cause T-cell proliferation response and was associated with partial restoration of microbiota composition [111]. Pouthidis et al. expanded upon the immune dysfunction associated with obesity, feeding a “fast food diet” that resulted in CD4+ Th17 biased immunity, changes in microbial composition, and abdominal obesity in mice. However, intervention with yogurt containing a mixture of S. thermophiles, *L. bulgaricus, L. acidophilus, B. bifidus, L. casei*, and *L rhamnosus* inhibited age-associated weight gain, while *L. reuteri* ATCC 6475 administered in drinking water prevented abdominal fat pathology independent of baseline diet. These effects were conferred with purified CD4+ T cells, and depended on active immune tolerance by the induction of Foxp3+ regulatory T cells and IL-10. [112]. These studies suggest that supplementation with *L. reuteri* restores a beneficial balance in the Th17/Treg host immunity, even in those individuals who have a pro-inflammatory immune state and chronic inflammation in association with a Westernized “fast food” diet. In mice fed a high fat and sucrose diet, epididymal fat mass and adipocyte size were significantly reduced with no difference in body weight [101]. As noted above, obesity is a disease characterized by disrupted microbiota, inflammation, and gut barrier alterations. The Gram-negative, strictly anaerobic bacteria, *Akkermansia muciniphila*, widely studied for its anti-inflammatory effects, has been shown to be in close proximity to the intestinal epithelium, supporting the hypothesis that *A. muciniphila* plays a role in mutualistic interactions between the gut microbiota and host that controls gut barrier functions as well as other physiological processes that occur in obesity [113]. *A. muciniphila* is a mucin-degrading bacterium that resides in the mucus layer of the gut, and is the dominant human bacterium that colonizes this nutrient-rich environment, representing anywhere from 3–5% of the microbial community in healthy individuals [114]. The abundance of this bacterium is inversely correlated with body weight in both humans and rodents. Everard et al. observed that viable *A. muciniphila* treatment for only four weeks reversed HFD-induced metabolic abnormalities including fat mass gain, metabolic endotoxemia, adipose tissue inflammation, and insulin resistance [115]. 

### 7.3. Multi-Strain Probiotics 

Several studies have investigated the effects of multi-strain probiotics, with the aim of determining their combinatorial efficacy compared to single-strain alternatives. For example administration of *L. curvatus* HY7601 mixed with *L. plantarum* KY1032 significantly reduced fat accumulation, and the combination showed a synergistic effect on inhibition of genes involved in fatty acid synthesis in mice fed a HFD after 9 weeks [116]. Further studies using *L. plantarum* KY1032 in combination with *L. curvatus* found that diet-induced obese mice treated with probiotics showed reduced body weight gain, fatty accumulation, and plasma insulin, leptin, total cholesterol, and liver toxicity biomarkers. These effects were associated with downregulation of pro-inflammatory genes in the liver, as well as upregulation of fatty acid oxidation genes (PGC1α, CPT1, CPT2, ACOX1) in the treatment group [84]. The effects of a large combination of strains were also examined. For example, intermittent administration of a mixture of 14 strains from several genera (*Lactobacillus*, *Lactococcus, Bifidobacterium, Propionibacterium, Acetobacter*) on monosodium glutamate (MSG)-induced obesity mouse model caused a significant reduction in the total body/visceral adipose tissue weight and improved insulin sensitivity compared to controls [117]. In a similar animal model, the same 14-strain multiprobiotic “Symbiter” was compared to administration of lyophilized monoprobiotics *B. animalis* VKL, *B. animalis* VKB, *L. casei* IMVB-7280, or a mix of all three strains. Supplementation with the mixture led to lower prevalence of obesity, reduced visceral adipose tissue (VAT) weight, and serum lipid levels compared to single strains. The authors purport that there are likely mutualistic interactions in the mixtures and therefore able to share with different metabolites, affect diverse receptors, and produce biologically-significant compounds that have synergistic effects [118]. Likewise, Alard and colleagues studied the combination of *L. rhamnosus* LMG S-28148 and *B. animalis subsp. Lactis* LMG P-28149 (mix) and found that HFD mice given the probiotic cocktail had significantly reduced body weight gain and adiposity. The authors noted that the observed reduced insulin resistance, and dyslipidemia could be explained by adipose tissue immune cell-remodeling, primarily affecting macrophages. At the gut level, there were changes in the uptake of fatty acids, restored expression of the short chain fatty acid receptor GPR43, and microbiotal changes which included recovered levels of *Akkermansia muciniphila*, increased *Rickenellacea*, and decreased *Lactobacillaceae*. When *B. animalis subsp lactis* LMG P-28149 was given alone, its efficiency was comparable to the mixture in regards to reduction in body weight, adipose tissue mass, and serum leptin levels [119]. Furthermore, administration of *B. longum* alone or mixed with *L. casei* Shirota reduced weight and triglycerides in HFD rats. Surprisingly, *B. longum* alone was better at modulating leptin level, fat mass, adipocyte size, lipoprotein lipase and PPAR-γ expression, and increasing adiponectin [120]. A recent study showed that a mixture of *B. lactis* Bi1, *B. breve* Bbr8, and *B. breve* BL10 (B. mix) was the best at ameliorating obesity in HFD mice compared to administration of single strains of LGG, *L acidophilus* LA1/K8, or alternative mixtures like *L. bulgaricus* LB2 with *S. termophilus* Z57. B. mix reduced weight gain, adipose tissue fat accumulation, adipocyte size, and macrophage/CD4+ T cell infiltration. Lastly, improvements in lipid profile and regulation of leptin and cytokine secretion were seen [121]. Overall, these studies showed that selective mixtures of probiotic strains could be more effective than single strains in ameliorating metabolic parameters associated with obesity.

## 8. Human Clinical Trials

Compared to experimental animal studies, human clinical trials that describe the effects of probiotics in obese individuals are sparse and controversial (Table 1). 

### 8.1. Lactic Acid Bacteria

Several strains of *Lactobacillus* have been tested in humans (Table 1). For example, the effects of *Lactobacillus rhamnosus* were assessed in pregnant women four weeks before their expected delivery date until six months postnatally. The children’s body mass index (BMI) was measured over the course of 10 years. When compared to children who were exposed to a placebo, it was found that the probiotic *L. rhamnosus* helped modulate the child’s weight gain during the first few years of life and during the initial phase of excessive weight gain but not later in life [122]. Other probiotics containing *Lactobacillus gasseri* SBT2055 and BNR17 species were administered to obese individuals over the course of 12 weeks [123,124]. *L. gasseri* SBT2055 lowered abdominal adiposity and body weight [123] while administration of *L. gasseri* BNR17 had no significant effects on reduction of weight or waist and hip circumference [124]. Several studies have examined the effects of the widely spread *Lactobacillus casei* (LcS) and *Lactobacillus paracasei* F19. In one study, individuals with metabolic syndrome were given LcS probiotic or a placebo for 12 weeks. After analyzing fecal samples with pyrosequencing of 16S rRNA genes, the results showed that LcS was unable to significantly influence the B/F ratio or ameliorate the gut barrier dysfunction [125]. Likewise administration of *Lactobacillus paracasei* F19 or flaxseed mucilage as control for six weeks to obese postmenopausal women had no effect on the metabolic markers [126]. A recent short, pilot study showed that *L. casei* strain Shirota given to 12 obese children for 6 months significantly reduced body weight in obese children, and increased HDL cholesterol. Additionally, there was a significant reduction in fecal concentrations of *Bifidobacterium* as well as *Bacteroides fragilis*; however, taken together, these studies showed that, overall, *L. casei* and *L. paracasei* F19 have limited efficacy in human subjects as a useful treatment for obesity and associated metabolic dysfunctions. 

Few studies have examined the effects of another probiotic bacterium with anti-microbial properties, *Lactobacillus reuteri*, that was shown to inhibit colonization of pathogenic microbes, remodel the commensal microbiota composition, reduce the production of pro-inflammatory cytokines, and strengthen the intestinal barrier. When *Lactobacillus reuteri* NCIMB 30242 was administered twice a day for six weeks to healthy, hypercholesterolemic adult men and women, there was no significant effect on BMI or body weight [127]. On the other hand, *Lactobacillus reuteri* JBD30I administered to overweight and obese individuals for 12 weeks had a positive effect by reducing dietary fat and free fatty acid absorption in the small intestine leading to increased excretion of the free fatty acids [128]. Although this may prove helpful in the treatment of obesity, thus far there are no clear and definitive results that support this theory. Several other *Lactobacillus* strains have been tested in humans with various outcomes (Table 1). For example, experiments where *Lactobacillus plantarum* A7 containing soy milk or regular soy milk was administered for eight weeks to patients with type 2 diabetes showed no significant differences in BMI or waist to hip ratio between the probiotic and placebo groups [129]. On the other hand, cheese-containing *Lactobacillus plantarum* TENSIA given to obese hypertensive patients for three weeks resulted in significant reduction of body weight, BMI, and fat mass of the subjects compared to subjects who consumed the control cheese [130]. Thus *Lactobacillus plantarum* TENSIA is among the few probiotics that has been shown to reduce body weight, BMI, and fat mass correlated to obesity in humans. Another *Lactobacillus* probiotic present in the gastrointestinal tract and tested for the treatment of obesity is *Lactobacillus salivarus* Ls-33. When administered to obese adolescents for twelve weeks *L. salivarius* was ineffective in exerting any impact on SCFA concentrations, a strong marker correlated to obesity or on the metabolic syndrome associated parameters. However, adolescents that consumed the probiotic *L. salivarius* had an increased ratio of the *Bacteroides-Prevotella-Poryphyromonas* group compared to the *Firmicutes* belonging bacterial group suggesting a modulatory role of *L. salivarius* on fecal microbiota composition [131,132,133]. However, supplementation with VSL#3^®^ in obese Latino adolescents increased adiposity with no significant detectable changes in gut microbiota, gut appetite-regulating hormones, liver fat and fibrosis and dietary intake [134]. Lastly, administration of the plant-derived lactic acid bacterium *Pediococcus pentosaceus* (LP28) that was heat-killed significantly lowered BMI, body fat, and waist circumference, whereas live LP28 and placebo exhibited no significant differences. Therefore, heat-killed LP28 may be helpful in the treatment of obesity, although more work needs to evaluate its efficacy as a valuable treatment option for obesity [135]. 

### 8.2. Lactobacillus and Bifidobacteria

One of the major probiotic that has been tested in human clinical trials is *Bifidobacterium* [60]. For example, when *Bifidobacterium lactis* HN019 was administered to patients with metabolic syndrome, there was an overall beneficial effect including reduction of obesity, blood lipids, and some inflammatory markers. Daily ingestion of probiotics resulted in a significant reduction in BMI, total cholesterol, and LDL compared to the control group [136]. In some clinical trials, *Bifidobacterium* was used in combination with other probiotic strains, including *Lactobacillus.* When a mixture of *Streptococcus thermophilus, Lactobacillus acidophilus, Bifidobacterium infantis, Bifidobacterium breve*, and *Enterococcus faecalis* was administered for 8 weeks as supplemented probiotic yogurt it significantly reduced body weight and BMI. This combination treatment may potentially be beneficial to those who suffer from metabolic syndromes. Since the supplemented probiotic yogurt contained several probiotics, it is unclear as to which probiotics or if all probiotic strains may contribute to improvement in obesity-related metabolic parameters [137]. Another probiotic combination containing *Lactobacillus gasseri* KS-13, *Bifidobacterium bifidum* G9-1, and *Bifidobacterium longum* MM2 administered twice a day for three weeks, resulted in a significant increase in lactic acid bacteria and Bifidobacteria. However, *Faecalibacterium prausnitzii*, a Firmicutes, was still more prevalent in the probiotic group compared to the placebo group resulting in an undesirable increased F/B ratio, but a beneficial reduction in the pro-inflammatory profile [138]. These results put into question how the contradictory effects weigh against each other phenotypically and, therefore, the efficacy of *Feacalibacterium prausnitzii* in the treatment of obesity remains unclear. Finally, a probiotic yogurt containing *Bifidobacterium lactis* Bb12 and *Lactobacillus acidophilus* La5 administered to subjects with non-alcoholic fatty liver disease (NAFLD) [139] and to overweight and obese women [140] had no significant effect on body weight and fat percentage compared to the control group. Other studies utilizing a mixture of *Lactobacillus* and *Bifidobacterium* have been more successful. For example, in a recent experiment, Gomes et al. administered *L. acidophilus, L. casei, L. lactis, B. bifidum*, and *B. lactis* to overweight and obese women on a dietary intervention for 8 weeks. Overall, supplementation with the probiotic mixture resulted in reduced abdominal adiposity, and increased antioxidant activity. Additionally, the dietary intervention plus probiotic supplementation produced a significant reduction in waist circumference, and plasma polyunsaturated fatty acids [141]. These results are consistent with a 2018 study demonstrating beneficial effects of administering multispecies probiotics from the genus *Bifidobacterium* and *Lactobacillus* (see table below) [142]. In this study involving 81 obese postmenopausal women, the probiotic mixture at both low and high doses positively modified glucose metabolism, lipid profile, waist circumference, visceral fat, serum uric acid levels, and LPS concentration. Additionally, serum total cholesterol, triglycerides, LDL cholesterol, glucose, insulin, and insulin resistance index were improved in the high dose group. Both groups had significant differences in fat percentage and visceral fat. Taken together these studies show that although some combination on *Lactobacillus* and *Bifidobacteria* probiotic strains may be more effective than others in ameliorating obesity-related metabolic defects, by and large, the effectiveness of probiotics on human obesity and their mechanisms of action are yet to be fully elucidated.

## 9. Animal vs. Clinical Trials

When comparing the efficacy of probiotics between animal and human studies it is clear that while the majority of animal studies show improved results on body weight, fat accumulation and other metabolic parameters, the results from human studies either failed to demonstrate such effects or are inconsistent at best. For example, while *Lactobacillus rhamnosus* GG has been shown to attenuate weight gain, reduce lipid accumulation, and reduce epididymal fat mass in mice fed a high fat diet, it was rather ineffective and did not prevent excessive weight gain, later in life, in pregnant women or their infants [122]. Similarly, *Lactobacillus gasseri* BNR17 reduced body weight gain and fat pad mass in animals while it had no effect on weight or waist/hip circumference in humans. Several factors may be responsible for the differences observed between animal and human studies. This includes the use of small cohorts in human trials, lack of long-term follow-up studies, and large differences in the probiotic strains used and their mechanistic variability. Therefore, it is desirable to identify and capitalize on unique and critical functional strengths of selective probiotic strains, characteristics that can be used to generate specialized consortia targeting specific pathways as intervention points in the application of probiotic therapy in obesity. Although an individual probiotic may not produce significant changes in body weight or fat loss, when used in combination with other strains targeting different pathways, a synergistic effect may emerge. The potential target pathways include regulation of fat absorption and excretion [143]; increase of primary cholic acids and activation of FXR receptor [144]; increase glucagon-like peptides GLP-1 and GLP-2 [145]; regulation of gene expression that lowers lipogenesis and increases beta oxidation of fatty acids (SREBP1c, ACAT, FAS, PPAR-α) [146]; regulation of ZO-1 and ZO-2 expression, leading to restoration of intestinal barrier function and decreased LPS absorption [147]; and increased synthesis of short chain fatty acids, especially butyric acid [148].

In addition to optimizing the experimental conditions using currently available probiotic strains and combinations, there are several “next-gen” probiotics under study for anti-obesity applications. Some of these candidates with novel mechanisms include but are not limited to *Saccharomyces cerevisiae* var. *Boulardii, Enterobacter halii*, and *Akkermansia muciniphila.* As mentioned earlier, *A. muciniphila* administration daily for 4 weeks to obese/diabetic mice reversed obesity, insulin resistance, and type 2 diabetes [115]. Additionally, *A. muciniphila* increases the mucus layer thickness in the gut back to a level comparable to lean mice and its levels were negatively correlated with gut permeability and inflammatory markers. Furthermore, *A. muciniphila* increased intestinal 2-oleoyglycerol, a bioactive lipid that stimulates secretion of GLPs. Although the data from *A. muciniphila* is encouraging, more work is required to elucidate the key mechanisms involved in its beneficial effects in human obesity [149]. 

Another next-generation probiotic, *Roseburia intestinalis*, metabolizes dietary fiber, is a major SCFA producer, provides energy for enterocytes, and has anti-inflammatory effects [150]. Cross-feeding chains have been established between *Bifidobacteria*, *F. prausnitzii*, and *R. intestinalis*, which enhance SCFA butyrate production in the colon. More work on the metabolic capabilities and cross-feeding chains in the gut could open novel avenues into the modulation of the composition and action of the microbiota. This knowledge coupled with more affordable profiling of individual gut microbial differences could lead to tailored efficacious therapies in the future [150].

The long-term benefits of probiotics in relation to the gut microbiome and obesity are poorly studied and their results are inconsistent. For example, in one study, pregnant women were fed a probiotic mixture containing *B. bifidum* W23, B. lactis W52, and Lc. Lactis W58 during their last six weeks of pregnancy. The composition of the microbiota in the children of these women was examined for the first six years of life, and the study showed that there were no long-lasting differences [151]. However, another study examined the long-term safety and benefits of perinatal administration of certain probiotics. The results showed that overall prenatal probiotic intervention was safe in the long term. In addition, children who regularly consumed probiotics had a decreased risk of being overweight in a long-term follow-up [152]. Thus, although long-term efficacy of probiotics are still controversial and more research is needed to establish their effects, probiotic products have been proven to be safe for human use. 

## 10. Conclusions and Perspectives

Obesity has far-reaching and burdensome consequences not only in terms of health outcomes for individuals, but also in exerting a significant financial impact on society at large. The clinical treatment of obesity has been met with immense challenges, related at least in part to the complexity of its pathophysiology in the context of biopsychosocial variability. Most of the previously approved medications for obesity have been removed from the market due to various adverse effects and the failure of obese individuals to maintain long-term weight loss. While interventions such as bariatric surgery can be effective in reducing excess weight for some individuals, the procedure is highly invasive, risks unforeseen complications, and requires a tremendous effort in adopting a new lifestyle. These realities beg the scientific and clinical community to develop novel approaches to address this ever-growing problem, and among the potential solutions, probiotics, which are generally considered safe for human health, have shown some promise.

The human gut plays host to trillions of bacteria, known collectively as the microbiota. This diverse ecosystem has evolved with us and is intricately linked to physiological processes that affect many organ systems including cardiovascular, neural, immune, and metabolic. Research over the last few decades has unlocked the door to understanding the role of the microbiota in energy homeostasis regulation, and how dysbiosis may be implicated in the pathophysiology of obesity through particular hormonal, neural, or metabolic mechanisms. Evidence thus far suggests that certain bacterial strains in a distinct equilibrium are associated with obesity, but which microbial community may be causally linked to obesity is still unknown. Animal and human studies have attempted to correct for gut dysbiosis by targeting the gut microbiota using probiotics but, given that this work is still in its early stages, there is limited data from which to draw deeper and clear meaningful conclusions beyond simple associations, which carries the risk of misinterpretation, or assigning excess value to expected results when translating animal protocols into human trials. 

Future investigation should attempt to understand how alterations of the gut microbiota lead to obesity or how obesity impacts changes in microbiome composition. This dualistic relationship and minute interactions between the host and flora, including genetic material exchange, may hold the key to meaningful clinical translation. Further understanding of this complex cross talk will aid in the development of more tailored and targeted implementation of probiotic therapies. Additionally, most of the aforementioned studies have been performed in tightly controlled animal models, which limit their potential application to human subjects, who will likely need to be stratified based on specific markers that take into account lifestyle, age, genetics, and other environmental influences on microbiota composition. Elucidation of the metagenomic relationship between changing microbiota and probiotic species under different diets/nutritional states will also be needed. Furthermore, most of the research in this dynamic field has been conducted using *Lactobacillus* and *Bifidobacterium* strains, thus creating the necessity for identifying new bacterial candidates along with their potential mechanistic effects on obesity. 

Thus far, clinical cohorts consisted of relatively small sample size, and focused on short-term physical parameters, or inflammatory markers, making long-term follow up studies highly desired in future work. Additional randomized placebo-controlled trials will help develop clinical guidelines for the use of probiotic therapy in obesity, and formulate nutritional recommendations and address safety concerns regarding functional foods that contain probiotics like fermented dairy products and kimchi. Questions about specific bacterial strains’ effect on microbial composition, duration of treatment, and appropriate dose are still left unanswered. Notwithstanding these shortcomings, there is no doubt that probiotic therapy represents an exciting new frontier in the treatment of obesity and associated metabolic dysfunctions.

## Figures and Tables

**Table 1:** Probiotics’ efficacy in animals vs. human clinical trials.

Strain	Animal Study Findings	Human Clinical Trials Findings	Reference
*Lactobacillus*			
*Lactobacillus rhamnosus*	*L. rhamnosus* GG attenuated weight gain, ↓lipid accumulation, ↓epididymal fat mass *L. rhamnosus* PL60 ↓body weight and ↓ white adipose tissue mass	*L. rhamnosus* GG modulated weight gain during the initial few years of excessive weight gain in life	[83] [84,100] [122]
*Lactobacillus gasseri* SBT2055 (LG2055)	Prevented body weight gain & fat accumulation. ↓mesenteric/retroperitoneal adipocyte size	Probiotic LG2055 ↓ abdominal adiposity and ↓ body weight. May improve metabolic disorders	[94,95] [123]
*Lactobacillus gasseri* BNR17	Attenuated ↑ in body weight and fat pad mass. ↓ body weight and white adipose tissue weight	No significant ↓ in weight or waist/hip circumference	[96,97] [124]
*Lactobacillus paracasei*	*L. paracasei* ST11 (NCC2461) attenuated weight gain and abdominal fat accumulation *L. paracasei* HII01 ↓ body weight and visceral fat	*L. paracasei* F19 showed no effect on metabolic	[98,99] [126]
*Lactobacillus reuteri*	*L. reuteri* ATCC 6475 ↓ weight gain and abdominal fat pathology	*L. reuteri* NCIMB 30242 showed no BMI or body weight differences *Lactobacillus reuteri* JBD30I ↓ dietary fat absorption and may be helpful in the treatment of obesity	[112], [127] [128]
*Lactobacillus plantarum*	PL62 ↓ body weight and adipose tissue mass (epididymal, inguinal, mesenteric, perineal) PLQ180 ↓ weight gain and epididymal fat weight Strain DK211 ↓ weight gain, ↓body fat accumulation, and ↓ organ weight Strain LG42 ↓ body weight, ↓ epididymal fat, ↓ back fat Strain TN8 improved body weight Strain LN4 ↓ body weight gain and ↓ epididymal fat mass	*Lactobacillus plantarum* A7 showed no difference in BMI or waist to hip ratio Heat killed LP28 ↓ BMI, ↓ body fat, and ↓ waist circumference. *Lactobacillus plantarum* TENSIA ↓ body weight, ↓ BMI, and ↓ fat mass	[72,87,88,89,90,91] [129,130], [135]
*Lactobacillus salivarius*	*L. salvarius* UCC118Bac(+)showed no improvement in metabolic profile ↑ Bacteroidetes, ↑ Proteobacteria, ↓ Actinobacteria	*L. salivarius* Ls-33 ↑ *Bacteroides-Prevotella-Porphyromonas* to *Firmicutes* ratio SCFA not changed *L. salivarius* not related to metabolic syndrome	[103] [133], [132]
*Lactobacillus casei* strain Shirota (LcS)	Body weight, BMI, fat mass, leptin, and glucose levels lower in HFD-LcS group compared to HFD rats.	Reduced fecal concentrations of *Bifidobacterium* as well as *Bacteroides fragilis*, *Atopobium cluster*, and *Lactobacillus gasseri*. There was a significant drop in body weight and an increase in HDL cholesterol.	[153], [154]

↓: decreased; ↑: increased.
